# Illumina-Based Analysis of Endophytic and Rhizosphere Bacterial Diversity of the Coastal Halophyte *Messerschmidia sibirica*

**DOI:** 10.3389/fmicb.2017.02288

**Published:** 2017-11-20

**Authors:** Xue-Ying Tian, Cheng-Sheng Zhang

**Affiliations:** Marine Agriculture Research Center, Tobacco Research Institute of Chinese Academy of Agricultural Sciences, Qingdao, China

**Keywords:** *Messerschmidia sibirica*, halophyte, endophytic bacteria, rhizosphere bacteria, diversity, Illumina HiSeq

## Abstract

Halophytes play important roles in coastal ecosystems. However, few reports have described bacterial communities related to halophytes, and the distribution patterns of these bacteria in different plant tissues have been rarely compared. This paper mainly studied the diversity and community structure of endophytic and rhizosphere (Rh) bacteria related to the halophyte *Messerschmidia sibirica*, a dominant species in the coastal zone of Shandong Peninsula, China. We collected leaf (Lf), stem (Sm), root (Rt), Rh, and bulk (Bl) control soil samples, and sequenced the V5–V7 region of the bacterial 16S rRNA gene using the Illumina HiSeq platform to identify bacterial communities originating from different plant habitats. We found that the bacterial richness and diversity in Rh were significantly higher than those in the leaves, Sm, and Rt, but lower than those of the Bl control soil. In total, 37 phyla and 438 genera were identified. Microbial-diversity analysis showed that *Proteobacteria* and *Actinobacteria* were the dominant phyla and that *Pseudomonas, Bacillus, Sphingomonas, Streptomyces, Microbacterium, Rhizobium*, and *Nocardioides* were the dominant genera. However, there were clear differences in community diversity and structure among the samples. Endophytic bacteria community in Lf, Sm, and Rt shared more similarity than those in Rh and Bl control soil. The numbers of operational taxonomic units exclusive to the Lf, stem, Rt, Rh, and Bl control soil samples were 51, 43, 122, 139, and 922, respectively, implying habitat-specific patterns. Principal coordinate analysis demonstrated differences were apparent in the bacterial communities associated with habitats. On the whole, *M. sibirica* affected bacterial diversity and structured the bacterial community. This study provides insight into the complex microbial compositions of coastal halophytes.

## Introduction

Along with emerging research on plant–microbe interactions, accumulating evidence suggests that endophytic and rhizosphere (Rh) bacteria play important roles in plant growth. These microbes can benefit plants by producing plant hormones (Etesami et al., 2015), enabling nutrient uptake (Silveira et al., 2016), increasing stress resistance (Yi et al., 2016; Shahzad et al., 2017), or altering the Rh microbial balance (Laanbroek et al., 2013; Chen et al., 2017). Moreover, the bacterial community and diversity vary across different plant species (Ramírez-Elías et al., 2014), colonization sites (Jin et al., 2014), and growing environments (Xu et al., 2014). Therefore, research into endophytic and Rh bacterial diversity is important for clarifying the functions of these bacteria and excavating these bio-resources. In recent years, most studies have focused on microbes associated with glycophytes or crop species (Xu et al., 2014; Haque et al., 2015; Majeed et al., 2015), while reports on bacterial communities associated with plants in extreme environments are limited.

The intertidal zone is an important part of the coastal environment, exhibiting characteristics of both marine and terrestrial ecosystems (Wiegert et al., 1981). Because of the characteristics of coastal environments (such as high salinity and tidal changes), a large number of halophytes grow there and perform important ecological functions to maintain the balance of the intertidal ecosystem. Halophytes have evolved various strategies to survive in this saline environment, and bacteria associated with halophytic plants may be key factors in such strategies. A large number of endophytic bacteria from halophytes have been shown the ability to improve salt tolerance of their host plants. For example, Navarro-Torre et al. (2017) reported that the endophytic bacteria from *Arthrocnemum macrostachyum* could improve its salt tolerance. Hashem et al. (2016) revealed that the growth promotion effect of endophytic bacteria and arbuscular mycorrhizal fungi (AMF) on *Acacia gerrardii* under salt stress were associated to a tripartite mutualistic symbiosis in *A. gerrardii*. Moreover, these microorganisms have been expected to be used in microbial-assisted phytoremediation of saline soils (Syranidou et al., 2016; Zhao et al., 2016). Different plant organs and the Rh may influence the abundances of bacteria and structures of bacterial communities, and it is therefore important to understand these parameters.

*Messerschmidia sibirica* is a salt-secreting halophyte that is widely distributed on the sandy beaches of northern coastal areas in China (Xiang et al., 2008). *M. sibirica* is a useful germplasm resource with high commercial value and ecological importance. For example, it can be used for environmental protection, such as sand stabilization, soil improvement, and phytoremediation (Jin et al., 2012). *M. sibirica* also has value in the medical, feed, and landscaping industries (Wang and Zheng, 2016). Consequently, research into the diversity of endophytic and Rh bacteria related to *M. sibirica* makes a great contribution to understand the connection between these microbes and the plant’s tolerance to high salt and infertile soil environments. They can also be potentially used in agricultural, biotechnological, and medical fields. It has been confirmed that endophytic bacteria from *M. sibirica* exhibited good plant growth-promoting ability (Shin et al., 2007). However, little information is available about the endophytic and Rh bacterial community of the plant. Thus, the objective of the present study was to obtain a broad overview of the bacterial community structures of different tissues and the Rh of *M. sibirica*, as well as the surrounding bare soil, using high-throughput sequencing. This represents the first study to characterize endophytic and Rh bacteria related to the halophyte *M. sibirica* in a Chinese coastal area. Our results provide new insight into this bacterial community and a foundation for future studies.

## Materials and Methods

### Sample Collection and Surface Sterilization

Sampling sites located in the Qingdao coastal zone (36°5′23″ S and 120°27′55″ E) in the Shandong Province of China. The local area has a temperate continental climate, possessing a mean air temperature of 13°C and an annual rainfall of 530–630 mm (Ma et al., 2013). *M. sibirica* is the dominant species in the intertidal zone. Three sampling sites of approximately 500 m each were selected, and three healthy plants were randomly collected from each site in July 2016. Sterilized gloves and spades were used to collect the samples, and the spades were sterilized between each collection. The mixture obtained from the three plants at the same sampling site constituted a sample. Samples collected included leaves (Lf), stems (Sm), roots (Rt), Rh soil, and bulk (Bl) control soil. The soil close to the Rt (0–3 mm from the Rt surface) was defined as the Rh soil (Zhang and Kong, 2014). Samples collected from locations 10–15 cm away from the Rt were considered as Bl control soil samples (Smalla et al., 2001). All samples were placed in aseptic bags which placed ice straightway and transported back to our lab. The plant materials (Lf, Sm, and Rt) were surface-sterilized according to Pereira and Castro (2014) method.

### DNA Extraction, PCR Amplification, and Gene Clone Library Construction

After the addition of liquid nitrogen, Lf, Sm, and Rt samples were frozen and ground rapidly to a fine powder in a sterilized and pre-cooled mortar, then transferred to a bead tube. For the Rh and Bl samples, 0.5 g of each sample was used for DNA extraction. Total DNA extraction was performed based on the instructions of the PowerSoil^®^ DNA Isolation Kit. DNA was stored at -20°C until subsequent analysis.

The target-specific primers 799F (5′-AACMGGATTAGATACCCKG-3′) and 1193R (5′-ACGTCATCCCCACCTTCC-3′), which do not amplify the chloroplast or mitochondrial 16S rRNA genes of *M. sibirica*, were used to amplify the V5–V7 region of the bacterial 16S rRNA gene (Beckers et al., 2016). Every PCR reaction was performed with Phusion^®^ High-Fidelity PCR Master Mix (New England BioLabs, Ipswich, MA, United States). Finally, sequencing and construction of the 16S rRNA gene clone libraries were performed at Novogene (Beijing, China) using the Illumina HiSeq 2500 platform, and 250-bp paired-end reads were created.

### Sequence Processing and Analysis

Paired-end reads were merged using FLASH software (v1.2.7; Magoč and Salzberg, 2011). Using QIIME (v1.7.0; Bais et al., 2006) processing and the UCHIME arithmetic (Edgar et al., 2011), and then obtained effective tags. Each operational taxonomic unit (OTU) was defined as a cluster of reads with 97% sequence identity. Each OTU was annotated with the SSU-rRNA SILVA database^1^ using MOTHUR (Wang et al., 2007). The complete sequences generated in this study are available in the NCBI SRA database under accession number SRR6161354 (Lf), SRR6161411 (Sm), SRR6161618 (Rt), SRR6161623 (Rh), and SRR6161637 (Bl).

The dataset without singletons was rarefied to the lowest number of reads (35,880) recovered from our samples for comparative analysis of species richness and diversity indices (Chao1, Shannon, Simpson, ACE, and good-coverage) among samples, using QIIME (v1.7.0; Kuczynski et al., 2011).

### Statistical Analysis

Statistical analyses were performed with the vegan package (Oksanen et al., 2011) in R (v2.15.3). One-way ANOVAs and Tukey–Kramer tests were used to test for differences in sample alpha diversities (observed OTUs and Shannon, Chao 1, and ACE richness and diversity indices). Heatmap images were created using the R package “heatmap” program (Kolde, 2012), and Venn diagrams were produced with the Venn-Diagram program (Chen, 2012). To explore variation in bacterial community compositions among samples, correspondence analysis (CA) was conducted in the R (v2.15.3) package ca (Nenadic and Greenacre, 2007). The relationships between bacterial community structures were evaluated by principal coordinate analysis (PCoA). Furthermore, we use LEfSe software (v1.0) to identify differentially abundant families among samples for biomarker discovery (Segata et al., 2011). Significant distinctions in the constituent of bacterial communities from the various habitats were determined with Adonis.

## Results

### Characteristics of Sample Sequence Tags

**FIGURE 1:**
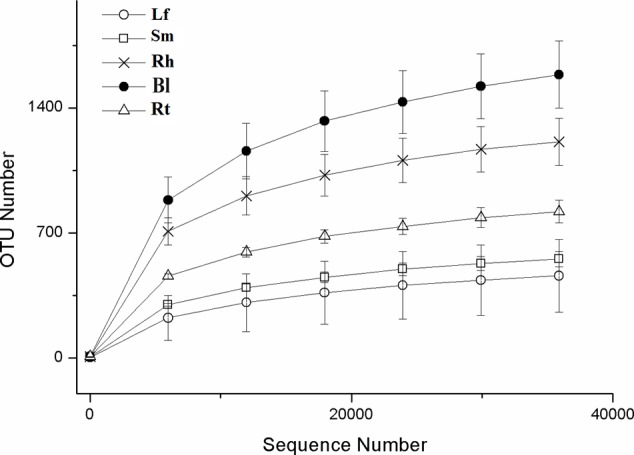
Rarefaction curves based on the sequences of the V4–V5 region of the 16S rRNA gene from samples associated with *M. sibirica*. Error bars represent the standard error of three replicates. Lf, leaf; Sm, stem; Rt, root; Rh, rhizosphere; Bl, bulk control soil.

**FIGURE 2:**
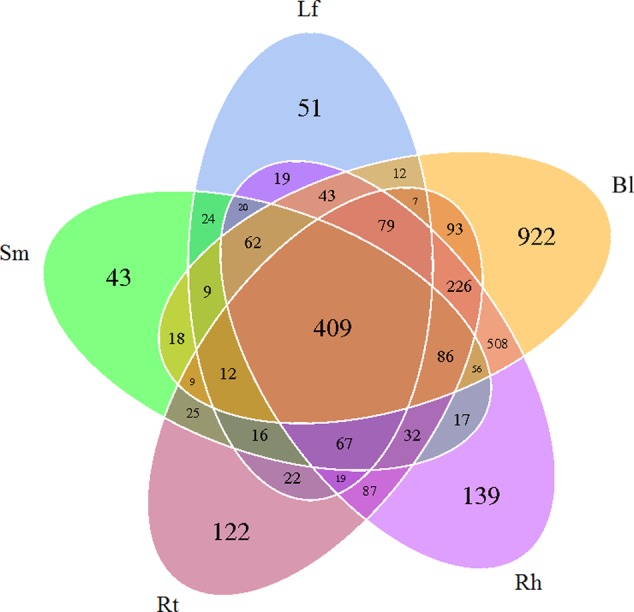
Venn diagram showing the OTUs shared among different samples associated with *M. sibirica.* Lf, leaf; Sm, stem; Rt, root; Rh, rhizosphere; Bl, bulk control soil.

After read-quality filtering, a total of 821,613 high-quality sequences were queried. The number of high-quality reads per sample ranged from 46,290 to 68,650 (average length of 376–378 bp) (Supplementary Table S1). Rarefaction curves (**Figure 1**), combined with the estimated coverage values (**Table 1**), suggested that the libraries were sufficiently large to capture a large majority of the bacterial diversity in the samples used in this study. Interestingly, the rarefaction curves indicated higher OTU numbers in the Rt samples than in the Lf and Sm samples, as well as higher numbers in the Bl control soil samples than in the Rh samples. A total of 3254 OTUs were detected across all libraries with 403 OTUs common to all samples (**Figure 2**). The numbers of OTUs exclusive to the Lf, Sm, Rt, Rh, and Bl samples were 51, 43, 122, 139, and 922, respectively. The shared bacterial OTUs mainly belonged to the *Proteobacteria* (229), *Actinobacteria* (127), *Firmicutes* (28), and *Bacteroidetes* (10) at the phylum level and *Rhizobium* (21), *Bacillus* (17), *Sphingomonas* (15), and *Streptomyces* (11) at the genus level.

**Table 1:** Operational taxonomic unit (OTU) richness and diversity indices of different samples associated with *M. sibirica* with a 97% similarity cut-off.

Sample name	Sample origin	OTUs observed	Shannon	Chao1	ACE	Coverage (%)
Lf	Leaf	527 ± 74 d	4.02 ± 0.65 d	465 ± 93 d	480 ± 92 d	99.7
Sm	Stem	553 ± 42 d	5.12 ± 0.29 c	671 ± 66 cd	666 ± 59 d	99.6
Rt	Root	821 ± 63 c	6.64 ± 0.19 b	979 ± 168 c	990 ± 144 c	99.5
Rh	Rhizosphere	1210 ± 188 b	7.90 ± 0.48 a	1351 ± 148 b	1375 ± 144 b	99.4
Bl	Bulk control soil	1587 ± 132 a	8.35 ± 0.61 a	1847 ± 234 a	1853 ± 220 a	99.0

*Values are the means of three replicates ± SD. Values within a column followed by different lowercase letters are significantly different (*P* < 0.05).*

### Microbial Community Richness and Diversity

The richness and diversity values of the bacterial communities after normalization are shown in **Table 1** and **Figure 1**. Bl samples harbored the highest number of OTUs. The Chao1 and ACE richness estimators and Shannon index were calculated based on a 3% genetic distance for all samples (**Table 1**), and all values were highest in the Bl samples, followed by Rh, Rt, Lf, and Sm.

### Microbial Taxonomic Analysis at the Phylum Level

**FIGURE 3:**
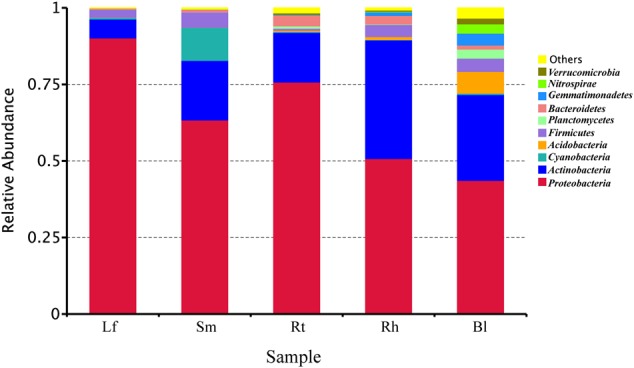
Relative abundances of bacteria at the phylum level in different samples. Lf, leaf; Sm, stem; Rt, root; Rh, rhizosphere; Bl, bulk control soil.

High-throughput sequencing revealed the diversity of bacterial communities in different samples at the phylum level (**Figure 3**). Thirty-seven phyla were identified, and the relative abundances of the top 10 phyla (each relative abundance, >1%) are shown in **Figure 3**. The phyla *Proteobacteria, Actinobacteria, Cyanobacteria, Acidobacteria, Firmicutes, Planctomycetes, Bacteroidetes, Gemmatimonadetes, Nitrospirae*, and *Verrucomicrobia* were detected in all samples; however, their relative abundances varied across different samples. *Proteobacteria* and *Actinobacteria* were the predominant bacterial phyla, accounting for more than 43.7 and 6.2%, respectively, of all samples. The abundance of *Cyanobacteria* was 10.8% in the Sm samples, which was much higher than that in the other samples. *Acidobacteria, Planctomycetes, Nitrospirae*, and *Verrucomicrobia* were higher in the Bl samples, while *Firmicutes* were lower in the Rt than in other samples.

**FIGURE 4:**
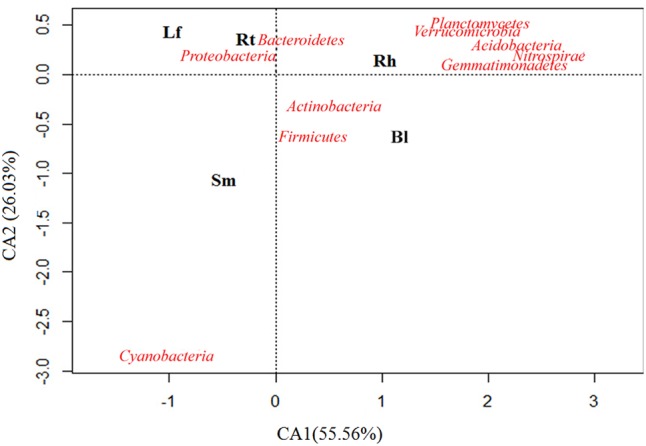
Correspondence analysis (CA) performed on the relative abundance of soil bacterial phyla of the Lf, Sm, Rt, Rh, and Bl control soil. The bacterial species is closer to a sample, which indicates these species were dominant species in this sample. The position of a sample in the multivariate space is defined using all the size classes: closeness reflects similar abundance and distance reflects scarcity.

Samples were scattered among the four quadrants in the CA analysis plot (**Figure 4**), suggesting the presence of different dominant bacterial communities in different samples, although the major bacterial communities appeared similar between the Lf and Rt samples. In addition, communities in the Rt and Rh samples were relatively similar compared to those in other organs or habitats, and this was due to the assignment of more sequences to the *Bacteroidetes* and *Proteobacteria* phyla. *Cyanobacteria* appeared along the edge of the graph, indicating that this phylum may have appeared sporadically or in a special habitat. Although CA provides only a broad resolution, it indicated a trend for different bacterial compositions in different habitats. For example, the enrichment of *Acidobacteria* in the Rh and Bl samples indicates that they appear to live in the Rh and Bl soils.

### Microbial Taxonomic Analysis at the Genus Level

**FIGURE 5:**
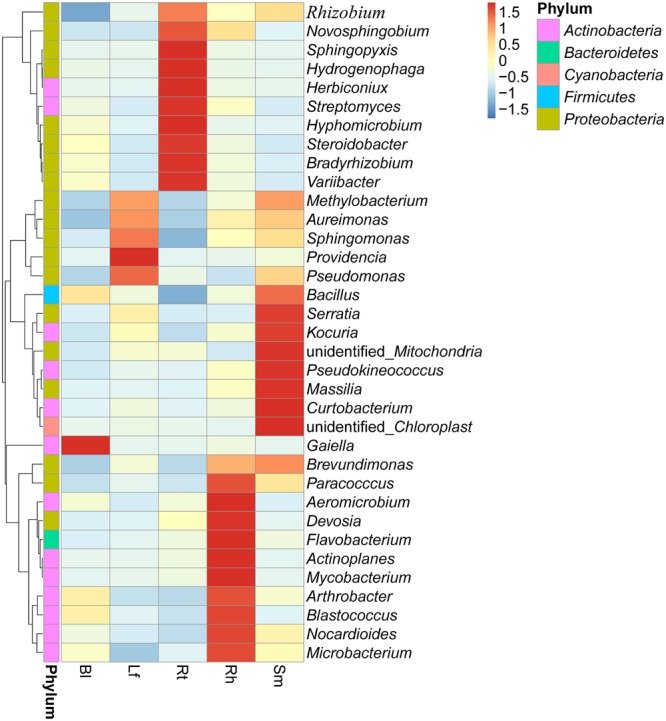
Heatmap displaying the relative abundances of the most dominant genera (top 35) in each sample. The dendrogram represents complete-linkage agglomerative clustering, based on Euclidean dissimilarities. Lf, leaf; Sm, stem; Rt, root; Rh, rhizosphere; Bl, bulk control soil.

Clustering of the top 35 genera is shown in **Figure 5** (supporting data are shown in Supplementary Table S2). These classified bacterial genera belonged to five phyla. Among these, 19 genera belonged to *Proteobacteria*, 13 to *Actinobacteria*, 1 to *Cyanobacteria*, 1 to *Firmicutes*, and 1 to *Bacteroidetes*. The following genera were the most abundant (>0.2%) across all samples: *Pseudomonas, Bacillus, Sphingomonas, Streptomyces, Microbacterium, Rhizobium*, and *Nocardioides*. However, species distributions differed greatly across different samples. *Methylobacterium, Aureimonas, Sphingomonas, Providencia*, and *Pseudomonas* were mainly distributed in the Lf, while *Bacillus, Derratia, Kocuria, Massilia*, and *Curtobacterium* were dominant in the Sm. Similarly, Rt samples possessed more species belonging to *Rhizobium, Novosphingobium, Sphingopyxis, Hydrogenophaga, Herbiconiux, Streptomyces, Hyphomicrobium, Steroidobacter, Bradyrhizobium*, and *Variibacter*. Dominant genera in the Rh included *Paracocccus, Aeromicrobium, Devosia, Flavobacterium, Actinoplanes, Mycobacterium, Arthrobacter, Blastococcus, Nocardioides*, and *Microbacterium*. Only one genus, *Gaiella*, was predominantly distributed in the Bl control soil. *Pseudomonas* was the dominant genus in Lf (25.6%) and Sm (17.65%) samples, while *Rhizobium, Actinoplanes*, and *Gaiella* were dominant in Rt (10.07%), Rh (5.79%), and Bl (3.71%) samples, respectively. The relative abundance of *Pseudomonas* was higher in Lf samples (25.6%) than in the other samples.

### Comparative Analysis of Bacteria in Different Sample Groups

**FIGURE 6:**
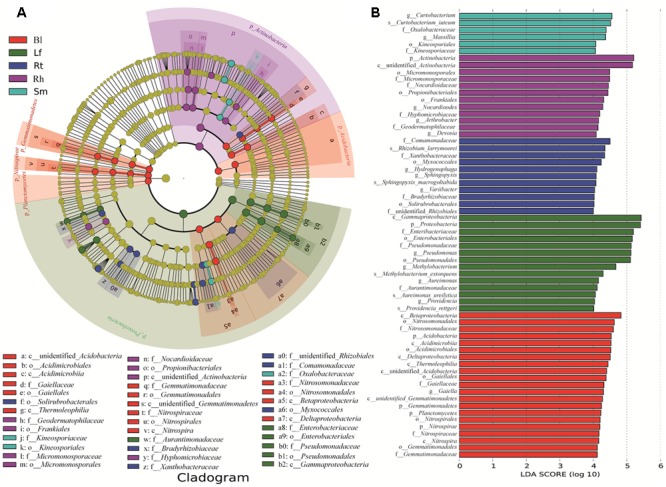
Groups from the phylum-to-family level determined to be significant representatives of their sample type, based on LefSe software analysis. **(A)** Cladogram representing the taxonomic hierarchical structure of the identified habitat biomarkers generated using LEfSe is shown. Each ring represents a taxonomic level, with phylum, class, order, and family emanating from the center to the periphery. Each circle is a taxonomic unit found in the dataset, with circles or nodes shown in color where the taxon represented a significantly more abundant group. **(B)** Identified biomarkers ranked by the effect size in different samples. The habitat biomarkers were identified as being significantly abundant (alpha value < 0.05) when compared among samples. Lf, leaf; Sm, stem; Rt, root; Rh, rhizosphere; Bl, bulk control soil.

Important distinctions were found in the compositions of bacterial communities in the five sample groups. Significantly different taxa abundances were found among the different samples, as determined by LEfSe (**Figure 6**). At the family level, *Pseudomonadaceae, Enterobacteriaceae*, and *Aurantimonadaceae* were significantly enriched in the Lf samples, while *Kineosporicaceae* and *Oxalobacteraceae* were more abundant in the Sm samples. *Bradyrhizobiaceae, Xanthobacteraceae*, and *Comamonadaceae* exhibited relatively higher abundances in the Rt samples. *Geodermatophilaceae, Nocardioidaceae*, and *Hyphomicrobiaceae* were abundant in the Rh samples. Four families showed significantly different abundances across samples: *Gaiellaceae, Gemmatimonadaceae, Nitrospiraceae*, and *Nitrosomonadaceae*. These differentially abundant taxa can be considered as potential biomarkers (LDA > 2, *P* < 0.05).

The bacterial communities differed according to the different organs and habitats of *M. sibirica*. To compare the species compositions observed in the Bl control soil, Rh, Rt, Sm, and Lf samples, Adonis was used to determine mean differences and their directionalities (sign) between two samples (**Table 2**). Based on these tests, a significant difference was observed between the Bl control soil and Sm samples (*R*^2^ = 0.55, *P* < 0.05), and Rt (*R*^2^ = 0.59, *P* < 0.05), as well as between the Rt and Lf (*R*^2^ = 0.38, *P* < 0.05), and Sm (*R*^2^ = 0.5, *P* < 0.05) samples. No significant difference was observed, however, between the Lf and Sm samples. The results showed that a large number of bacteria inhabited the Rh, Bl control soil, and Rt samples compared to that in the Lf and Sm samples.

**Table 2:** Adonis analysis of the difference among samples associated with *M. sibirica.*

Habitats	Leaf	Stem	Root	Rhizosphere
Bulk control soil	*R*^2^ = 0.43, *P* = 0.1	*R*^2^ = 0.55, *P* = 0.013	*R*^2^ = 0.59, *P* = 0.0013	*R*^2^ = 0.49, *P* = 0.1
Rhizosphere	*R*^2^ = 0.36, *P* = 0.1	*R*^2^ = 0.41, *P* = 0.1	*R*^2^ = 0.55, *P* = 0.1	
Root	*R*^2^ = 0.38, *P* = 0.0013	*R*^2^ = 0.50, *P* = 0.0013		
Stem	*R*^2^ = 0.16, *P* = 0.7014			

*The larger the value of *R*^*2*^ (the ratio of group variance to total variance), the more significant the differences were among the organs and habitats. *P* < 0.05 indicates a high reliability of the test.*

**FIGURE 7:**
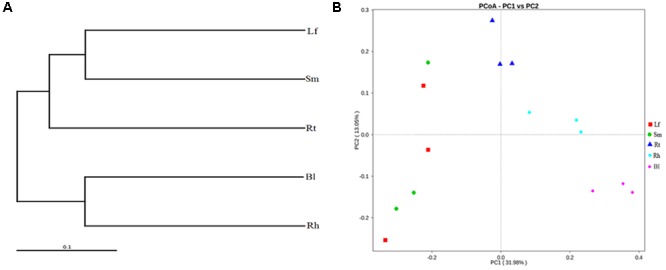
Cluster pedigree diagram **(A)** and principal coordinate analysis (PCoA) **(B)** of different microbiota in different samples based on weighted UniFrac distances. Lf, leaf; Sm, stem; Rt, root; Rh, rhizosphere; Bl, bulk control soil.

Beta-diversity analysis based on Unweighted Pair Group Method with Arithmetic Mean (UPGMA) clustering (**Figure 7A**) and PCoA (**Figure 7B**) were performed to compare the microbial compositions of different samples. Consistent with the results of the Adonis analysis, two different clusters were observed at the genus level in the UPGMA tree: group 1 consisted of the Lf, Sm, and Rt samples, and group 2 consisted of the Rh and Bl samples. This indicates that the microbiota of the Lf samples was more similar to those of the Sm and Rt samples than to other samples. Similarly, the Bl and Rh samples exhibited more similar bacterial compositions versus the Bl and Lf samples. PCoA revealed the main variations in bacterial community composition among the samples. The highest variations in the microbiota of different samples were 31.98% (PC1) and 13.05% (PC2), representing a strong separation based on the plant organs or habitats. Samples of the Rt, Rh, and Bl control soil from different *M. sibirica* plants clustered together, but the other samples showed some separation.

## Discussion

Based on OTU analysis and the Chao1, ACE, and Shannon’s diversity indices, the species diversity and richness were higher in the Rt of *M. sibirica* than in the Sm as well as Lf (*P* < 0.05, **Table 1**). Similar results were also reported for other plants, including *Oryza sativa* (Mano et al., 2007) and the halophyte *Phragmites australis* (Ma et al., 2013). Therefore, it could be concluded that the endophytic bacteria distribution varied greatly in different plant tissues, and the Rt hide more bacteria communities than Lf or Sm. Since most endophytic bacteria are from soil origin (Lamb et al., 1996; Hallmann et al., 1999), largest endophytic bacteria diversity in Rt may be attributed to the primary site of interaction between plants and soil (Hardoim et al., 2011). Meanwhile, the lower diversity in Lf may be related to the relatively low diversity of bacteria in the phyllosphere (Lindow and Brandl, 2003), as some endophytic bacteria in Lf may be derived from phyllosphere (Mano et al., 2007).

Previous data have verified that bacterial communities in the Rh exhibit higher richness than endophytes in the organs of halophytes (Szymańska et al., 2016; Tan et al., 2017). Consistent with these findings, the bacterial species diversity and richness in Rh soil samples of *M. sibirica* were greater than Rt samples. This difference can be explained partly by the environmental variability. Rh are rich in carbon sources secreted by plants, which can be used by bacteria (Wawrik et al., 2005), while Rt represent a relative stable niche for microorganisms. We also found that Bl control soil samples, which contained 33.8% of the total number of OTUs, showed higher diversity and richness than the samples associated with *M. sibirica*. Gomes et al. (2014) reported similar results, in which OTU richness was higher in the Bl soil than in the Rh of two mangroves, *Laguncularia racemosa* and *Avicennia schaueriana*. This effect of reduced bacterial richness in the plant Rh (compared to the Bl control soil) has been previously described as the “rhizosphere effect” (Sørensen et al., 1997). However, there are some reports contrary to the above findings. Yang et al. (2017) observed that rhizospheric samples of maize were more diverse than Bl soil samples. Similar results were also obtained in some halophytes (Fahmy and Ai-Thani, 2008). These different results indicated that the local environment and plant species had complex impacts on bacteria community.

Cluster analysis showed that bacterial communities varied across the different habitats. The Bl control soil, Rh, Rt, Sm, and Lf of *M. sibirica* were colonized by the same microbial families, but at different proportions. *Proteobacteria, Actinobacteria, Firmicutes*, and *Bacteroidetes* were most abundant phyla for endophytic and Rh bacteria (**Figure 3**), in agreement of previous studies about other plants with culture-independent (Jin et al., 2014) and culture-dependent methods (Szymańska et al., 2016). However, *Bacteroidetes* and *Proteobacteria* mainly appeared in Lf and Rt samples, while *Actinobacteria* and *Firmicutes* were dominant in the Rh and Bl control soil samples, indicating the presence of habitat specificity (**Figure 4**). Sequences assigned to *Actinobacteria* were more abundant in Rh-associated communities than in endophytic communities, which reflects a pattern noted in previous studies (Rastogi et al., 2012; Bodenhausen et al., 2013). In addition, the dominant phyla *Actinobacteria* and *Firmicutes* might play important roles in the ecology of *M. sibirica*, as these phyla are also common in other halophytes (Mora-Ruiz et al., 2015; Shi et al., 2015). Therefore, it is not surprising that relatively high abundances of *Actinobacteria* were found across all organs of *M. sibirica* and in the Rh and Bl control soil. Interestingly, *Acidobacteria* was relatively abundant in Bl control soil, even though this zone was alkaline, demonstrating that *Acidobacteria* colonization was not limited to acidic soil. This conclusion was also drawn by Xiong et al. (2012).

Among the top 35 genera, the dominant endophytes belonged to *Rhizobium, Streptomyces*, and *Pseudomonas*, many species of which have been reported to have beneficial effects for C or N cycling in the soil (Herridge, 2013). In addition, *Methylobacterium, Sphingomonas*, and *Actinoplanes* were common, and these play a role in plant–microbe interactions in halophytic ecosystems (Omer et al., 2004; Solans et al., 2011; Li et al., 2013). The *Methylobacterium* genus has been implicated in plant growth and shown effective in promoting seed germination (Omer et al., 2004). This genus also participates in plant N metabolism since most of these bacteria are capable of N fixation (Raja et al., 2006). Above all, we speculate that many beneficial microorganisms are present in *M. sibirica*, but the specific interactions between the bacteria and their host require further investigation.

Our results suggest that *M. sibirica* drove differentiation of the bacterial community structure. As verified by Adonis, significant distinctions occurred in the bacterial communities of various samples, with the exception of the Lf and Sm (**Table 2**). Compared to those in the Rh and Bl control soil, endophytic bacteria of the Lf, Sm, and Rt of *M. sibirica* shared more similarity (**Figure 7A**). It suggested that the endophytic bacteria of Lf, Sm, and Rt samples may share the same origin as mentioned above. The more endophytic bacterial similarity between the Lf and Sm may be related to some strains deriving from phylloplane and *caulosphere* as revealed in other studies (Mano et al., 2007). Slightly different from our results, Jin et al. (2014) reported that Lf and Sm samples of *Stellera chamaejasme* were clustered together, but were different from those of Rh and Rt. As demonstrated by PCoA, habitats explained 31.98% of the variation in the community structure, while sampling zones explained 13.05% (**Figure 7B**). Therefore, data from this study and previous investigations (Ma et al., 2013; Szymańska et al., 2016) indicate that *M. sibirica* and its tissues have a greater influence than sampling area for bacterial communities.

## Conclusion

This study is the first to elucidate the bacterial diversity and composition of the coastal halophyte *M. sibirica* using high-throughput sequencing methods. The dominant bacteria associated with *M. sibirica* were *Proteobacteria, Actinobacteria*, and *Firmicutes*. We demonstrated that the bacterial communities varied across the different habitats of Lf, Sm, Rt, the Rh, and Bl control soil. Our results showed that *M. sibirica* affected bacterial diversity and structured the bacterial community. These results provide insight into the complex microbial compositions of coastal halophytes. Further studies are necessary to elucidate the functional roles of these bacterial species in plant–microbe interactions in the inter-tidal zone, for example, how they may enhance or impair plant fitness.

## Author Contributions

C-SZ contributed to the conception of the study and wrote the manuscript. X-YT performed the experiments and data analyses.

## Conflict of Interest Statement

The authors declare that the research was conducted in the absence of any commercial or financial relationships that could be construed as a potential conflict of interest.

## Footnotes

1 http://www.arb-silva.de/
