# Supplementary material for: Illumina-Based Analysis of Endophytic and Rhizosphere Bacterial Diversity of the Coastal Halophyte Messerschmidia sibirica
Source: Front Microbiol. 2017 Nov 20;8:2288. doi: 10.3389/fmicb.2017.02288 (PMC5701997; doi:10.3389/fmicb.2017.02288)
Supplement: Supplementary file 1 [file Table_1.DOCX]

Table S1. Characteristics of effective tags from samples of endophytic bacteria and rhizosphere bacteria associated with *M. sibirica*.

| Sample | Sample origin | Sample site | Number of tags | Total length (bp) | Average length (bp) | Effective % |
| --- | --- | --- | --- | --- | --- | --- |
| Rh1 | rhizosphere | 1 | 68,650 | 25,931,114 | 378 | 86.74 |
| Rh2 | rhizosphere | 2 | 62,399 | 23,585,999 | 378 | 87.38 |
| Rh3 | rhizosphere | 3 | 53,392 | 20,163,681 | 378 | 80.78 |
| Lf1 | leaf | 1 | 48,795 | 18,375,411 | 377 | 71.51 |
| Lf2 | leaf | 2 | 54,720 | 20,619,165 | 377 | 85.41 |
| Lf3 | leaf | 3 | 58,901 | 22,230,593 | 377 | 79.27 |
| Rt1 | root | 1 | 44,461 | 16,794,173 | 378 | 66.46 |
| Rt2 | root | 2 | 59,302 | 22,384,532 | 377 | 81.54 |
| Rt3 | root | 3 | 51,932 | 19,613,472 | 378 | 79.5 |
| Bl1 | bulk control soil | 1 | 46,290 | 17,460,964 | 377 | 70.56 |
| Bl2 | bulk control soil | 2 | 53,373 | 20,132,902 | 377 | 72.88 |
| Bl3 | bulk control soil | 3 | 53,360 | 20,109,754 | 377 | 73.4 |
| Sm1 | stem | 1 | 57,660 | 21,688,138 | 376 | 79.13 |
| Sm2 | stem | 2 | 59,066 | 22,263,376 | 377 | 76.83 |
| Sm3 | stem | 3 | 49,312 | 18,569,229 | 377 | 64.41 |
