# Supplementary material for: Illumina-Based Analysis of Endophytic and Rhizosphere Bacterial Diversity of the Coastal Halophyte Messerschmidia sibirica
Source: Front Microbiol. 2017 Nov 20;8:2288. doi: 10.3389/fmicb.2017.02288 (PMC5701997; doi:10.3389/fmicb.2017.02288)
Supplement: Supplementary file 2 [file Table_2.DOCX]

Table S2. The relative abundances of the most dominant genera (top 35) in each sample.

| Taxon | Bl | Lf | Rt | Rh | Sm |
| --- | --- | --- | --- | --- | --- |
| Pseudomonas | 0.29 | 25.60 | 6.01 | 2.12 | 17.65 |
| Bacillus | 2.10 | 1.37 | 0.46 | 1.41 | 2.91 |
| Sphingomonas | 2.97 | 10.21 | 0.70 | 5.44 | 7.60 |
| Streptomyces | 1.03 | 0.29 | 5.83 | 1.71 | 0.25 |
| Actinoplanes | 0.18 | 0.12 | 0.47 | 5.79 | 0.04 |
| Providencia | 0.00 | 2.09 | 0.01 | 0.06 | 0.22 |
| Microbacterium | 1.90 | 1.26 | 1.60 | 2.99 | 2.00 |
| Rhizobium | 0.24 | 3.69 | 10.07 | 5.26 | 7.50 |
| Nocardioides | 0.99 | 0.51 | 0.27 | 3.95 | 1.80 |
| Sphingopyxis | 0.05 | 0.07 | 2.69 | 0.17 | 0.05 |
| Brevundimonas | 0.05 | 0.51 | 0.11 | 1.21 | 1.37 |
| Aeromicrobium | 0.49 | 0.15 | 0.43 | 1.99 | 0.17 |
| Gaiella | 3.71 | 0.01 | 0.02 | 0.26 | 0.01 |
| Serratia | 0.02 | 0.90 | 0.00 | 0.01 | 2.27 |
| Methylobacterium | 0.04 | 8.26 | 0.30 | 2.96 | 8.25 |
| Bradyrhizobium | 0.33 | 0.03 | 1.29 | 0.25 | 0.07 |
| Curtobacterium | 0.03 | 0.80 | 0.08 | 0.81 | 6.69 |
| Kocuria | 0.08 | 0.46 | 0.02 | 0.36 | 1.25 |
| Novosphingobium | 0.06 | 0.06 | 1.99 | 1.16 | 0.17 |
| Hydrogenophaga | 0.13 | 0.03 | 3.15 | 0.15 | 0.06 |
| Aureimonas | 0.02 | 2.64 | 0.11 | 1.56 | 2.15 |
| Flavobacterium | 0.01 | 0.05 | 0.18 | 1.42 | 0.19 |
| Steroidobacter | 0.42 | 0.04 | 1.41 | 0.25 | 0.02 |
| Pseudokineococcus | 0.00 | 0.17 | 0.10 | 0.45 | 1.71 |
| Mycobacterium | 0.17 | 0.21 | 0.27 | 1.61 | 0.18 |
| Variibacter | 0.61 | 0.03 | 2.38 | 0.39 | 0.06 |
| unidentified_Chloroplast | 0.30 | 0.53 | 0.25 | 0.04 | 10.75 |
| Paracocccus | 0.03 | 0.20 | 0.06 | 1.23 | 0.67 |
| Arthrobacter | 1.49 | 0.16 | 0.09 | 2.98 | 0.95 |
| unidentified_Mitochondria | 0.00 | 0.28 | 0.26 | 0.01 | 1.24 |
| Massilia | 0.05 | 0.14 | 0.02 | 1.03 | 4.34 |
| Hyphomicrobium | 0.27 | 0.01 | 1.37 | 0.10 | 0.03 |
| Devosia | 0.08 | 0.09 | 0.75 | 2.61 | 0.18 |
| Herbiconiux | 0.06 | 0.02 | 1.54 | 0.11 | 0.06 |
| Blastococcus | 0.79 | 0.18 | 0.02 | 1.78 | 0.16 |

Lf, leaf; Sm, stem; Rt, root; Rh, rhizosphere; NR, non-rhizosphere.
